# Restoring immune homeostasis in the spinal microenvironment: targeting mechano-inflammation and immunometabolic reprogramming

**DOI:** 10.3389/fimmu.2026.1806060

**Published:** 2026-06-19

**Authors:** Zhichao Chen, Junfeng Zeng, Zhiwei Zhang, Zhi Zheng

**Affiliations:** Department of Orthopedics, Chengdu Integrated TCM&Western Medicine Hospital, Chengdu, China

**Keywords:** axial spondyloarthritis, epigenetic memory, immunometabolism, intervertebral disc degeneration, spinal microenvironment

## Abstract

Chronic inflammatory diseases of the spine, typified by axial spondyloarthritis (axSpA) and intervertebral disc degeneration (IDD), impose a substantial burden through refractory pain and irreversible structural remodelling (pathological ossification or fibrosis). Although current anti-inflammatory therapies can alleviate symptoms, their capacity to arrest structural deterioration remains limited, underscoring the constraints of approaches that target inflammatory cytokines in isolation. This therapeutic intractability is rooted in the distinctive spinal microenvironment: high mechanical loading at the enthesis and the hypoxic niche of the intervertebral disc (IVD), through mechano-inflammatory coupling and maladaptive metabolic adaptation, actively drive pathological reprogramming of immune cells and create a barrier to the restoration of homeostasis. In this Review, we dissect the multidimensional crosstalk among biomechanics, metabolism and immunity, and delineate the central roles of Human leukocyte antigen B27 (HLA-B27) associated stress, metabolic reprogramming, and osteo-immune crosstalk in sustaining chronic inflammation. On this basis, we propose a paradigm shift from suppressing downstream mediators to modulating upstream microenvironments. We suggest that targeting metabolic checkpoints, interrupting mechanotransduction, and applying epigenetic interventions may promote inflammatory resolution and reset matrix homeostasis, thereby offering new strategies to restore spinal immune equilibrium and prevent structural failure.

## 1 Introduction

Chronic inflammatory spinal disorders, represented by axial spondyloarthritis (axSpA) and intervertebral disc degeneration (IDD), are major causes of disability worldwide. Based on the ASAS criteria, axSpA encompasses a broad spectrum from non-radiographic axSpA (nr-axSpA; active inflammation) to radiographic axSpA (r-axSpA, or classic AS), which features irreversible pathological ossification. The global prevalence of axSpA in adults is approximately 0.1%–1.4%. The disease typically affects young adults, leading to substantial functional impairment and a marked decline in quality of life (1, 2).

Although axSpA and IDD have distinct etiological origins, they share several key microenvironmental features during disease progression because of the unique anatomical and physiological constraints of the spine. axSpA is a systemic, HLA-B27 associated, immune-mediated disease, with enthesitis as its pathological hallmark, whereas IDD is primarily a local degenerative process. Nevertheless, both conditions involve mechanical loading sensed by Piezo1, local hypoxia, and activation of the NF-κB/NLRP3 inflammatory pathways. These shared drivers ultimately lead to opposite patterns of structural remodeling: pathological new bone formation in AS (r-axSpA), involving HLA-B27/unfolded protein response signaling, the IL-23/IL-17 axis, and activation of resident immune cells such as γδ T cells, MAIT cells, and ILC3s; and progressive matrix degradation in IDD, characterized by nucleus pulposus cell senescence, cartilaginous endplate degeneration, and Modic changes. This divergence from partially shared microenvironmental mechanisms represents a central paradox in spinal pathology (3–5).

The past two decades have witnessed a paradigm shift in the management of spinal diseases. Biologic disease-modifying antirheumatic drugs (bDMARDs) targeting TNF and IL-17A, together with JAK inhibitors, have transformed the clinical treatment of axSpA. Clinical evidence indicates that these therapies not only improve symptoms but may also partially slow radiographic progression through sustained control of inflammation (6, 7). However, several challenges remain in clinical practice, including inadequate response in a subset of patients, limited drug persistence, and residual structural progression despite achievement of inflammatory targets (8–10). Similarly, treatment options for IDD remain largely restricted to symptomatic analgesia or late-stage surgical intervention. No approved disease-modifying osteoarthritis drugs (DMOADs) are currently available to reverse the degenerative cascade or restore biomechanical homeostasis of the intervertebral disc (11).These therapeutic limitations suggest that the spine should not be regarded merely as a mechanical scaffold, but also as an “immune organ” with distinct anatomical constraints and physiological properties. Unlike highly vascularized tissues such as synovial joints, the spinal microenvironment is defined by highly specialized niches. First, the enthesis, as a stress-concentrating anchoring site, exhibits a unique mechanism of mechano-inflammatory coupling. Resident innate immune cells, such as γδ T cells, can be directly activated by mechanical loading and initiate sterile inflammation without exogenous pathogens (12–14). Second, the intervertebral disc, the largest avascular organ in the human body, contains a nucleus pulposus exposed to severe hypoxia, acidity, and hyperosmolarity. This harsh metabolic environment may force infiltrating immune cells to undergo metabolic reprogramming, locking them into a pro-inflammatory phenotype and thereby establishing a metabolic barrier that impairs inflammation resolution (15, 16).

This raises a central clinical paradox in the spinal microenvironment: why do similar mechanical stresses and inflammatory triggers drive osteogenesis and ankylosis at the enthesis, but promote resorption and degeneration within the IVD (17)? In this review, we propose a new theoretical framework: these opposing pathological outcomes arise from differential decoding of common signals by local spinal microenvironments, governed by lineage-specific epigenetic presets. We focus on how mesoderm-derived entheseal mesenchymal cells and notochord-derived nucleus pulposus cells act as distinct effector populations with different chromatin landscapes and developmental memories, leading to divergent cell fates under the same upstream signals, such as mechanosensitive Yes-associated protein (YAP) and transcriptional co-activator with PDZ-binding motif (TAZ) signaling. By dissecting this context-dependent signaling, this review aims to clarify how remodeling the local microenvironment, rather than simply suppressing cytokines, may break the vicious cycle of inflammation and provide mechanism-based therapeutic strategies to both prevent ankylosis and reverse degeneration.

## 2 Anatomical determinants of the spinal microenvironment and immune dysregulation

**Figure 1:**
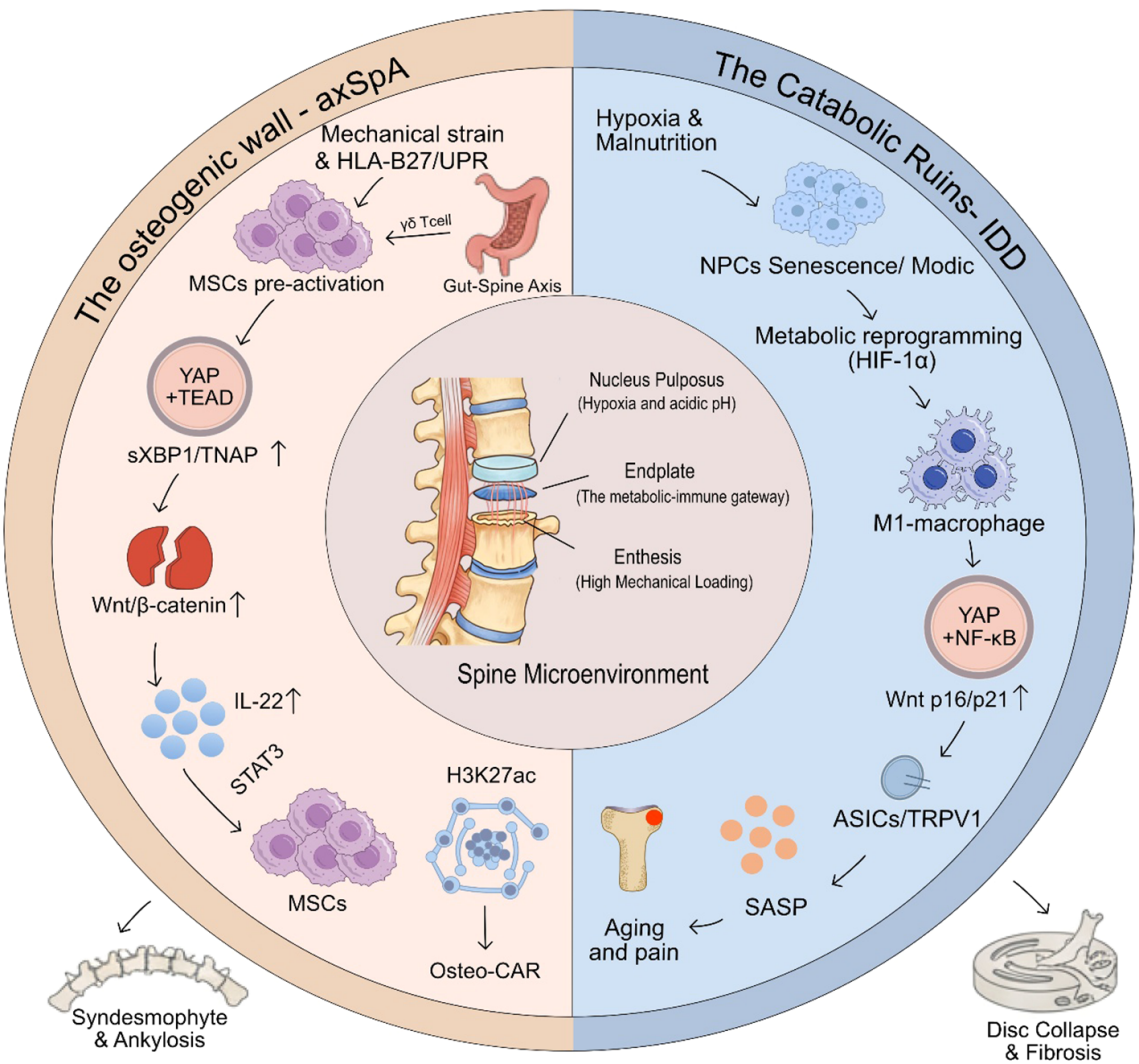
Mechanometabolic niches driving divergent structural fates in axSpA/AS and IDD. The spinal microenvironment (enthesis, nucleus pulposus, and endplate) dictates distinct pathological outcomes. Left (AS): Mechanical strain and HLA-B27-induced UPR activate the sXBP1-TNAP axis and H3K27ac-mediated super-enhancers, driving autonomous bone formation. Right (IDD): Hypoxia and malnutrition induce NPCs senescence, metabolic reprogramming (HIF-1α), and M1-macrophage polarization, leading to YAP/NF-*κ*B activation and SASP-mediated matrix degradation. This dichotomy illustrates how lineage-specific niches decode universal mechanoinflammatory signals into either pathological construction (AS) or destruction (IDD). axSpA, Axial Spondyloarthritis; AS, Ankylosing Spondylitis; IDD, Intervertebral Disc Degeneration; MSCs, Mesenchymal Stem Cells; HLA, Human Leukocyte Antigen; UPR, Unfolded Protein Response; sXBP1, Spliced X-box-binding protein 1; TNAP, Tissue-Nonspecific Alkaline Phosphatase; H3K27ac, Histone H3 lysine 27 acetylation; NPCs, Nucleus Pulposus Cells; HIF-1*α*, Hypoxia-Inducible Factor 1*α*; YAP, Yes-Associated Protein; TEAD, Transcriptional Enhancer Associate Domain; NF-*κ*B, Nuclear Factor-*κ*B; SASP, Senescence-Associated Secretory Phenotype; ASICs, Acid-Sensing Ion Channels; TRPV1, Transient Receptor Potential Vanilloid 1; Osteo-CAR, Osteogenic-C-X-C motif chemokine ligand 12-abundant reticular cells; STAT3, Signal transducer and activator of transcription 3.

Immune homeostasis in the spine is profoundly shaped by the architectural features of its local microenvironment. To explain why anti-inflammatory therapy often fails to restore tissue homeostasis, we must deconstruct three key anatomical dimensions that define pathological trajectories in the spine: the load-bearing enthesis, the IVD as an avascular and nutrient-deprived niche and the cartilage endplate (CEP) as an immunometabolic gatekeeping hub. Through their distinct biomechanical contexts, stringent metabolic constraints, and barrier functions, these sites each establish specialized niches that sustain chronic inflammation (**Figure 1**).

### 2.1 Enthesis: a mechanosensitive inflammatory niche that initiates inflammation

As the anatomical anchor connecting tendons and ligaments to bone, the enthesis is now regarded as a highly specialized “immuno-mechanical organ”. This concept extends beyond classical anatomy by emphasizing the synovio-entheseal complex-encompassing fibrocartilage, bursae, fat pads, and adjacent bone marrow-whose primary physiological role is to buffer and dissipate high mechanical stress. In pathological settings, however, this microenvironment becomes a focal point for converting physical strain into inflammatory cascades, a process termed mechano-inflammatory coupling (18).

Although immune-cell infiltration is commonly viewed as a hallmark of inflammatory flare, accumulating evidence suggests that, in the pathogenesis of axSpA, tissue-resident stromal cells are the initiators of early inflammatory programmes. Mesenchymal stem/stromal cells (MSCs) and fibroblasts at the enthesis constitutively express high levels of mechanosensitive ion channels, notably Piezo1. Upon exposure to pathological stretch or repetitive micro-injury, these channels sense changes in membrane tension and mediate rapid Ca^2+^ influx (19, 20). Studies have shown that, within the entheseal niche of axSpA, Piezo1 promotes pathological new bone formation through CaMKII signaling and acts synergistically with TNF-α and IL-17A to sustain entheseal inflammation. In the microenvironment of IDD, however, the same Piezo1 activation primarily induces intracellular calcium dysregulation, mitochondrial dysfunction, NLRP3 inflammasome activation, and YAP/TAZ-mediated extracellular matrix (ECM) degradation, thereby establishing a self-amplifying cycle between mechanical stress and inflammation (21). This burst of intracellular calcium signalling, acting as a pivotal second messenger, swiftly activates MAPK/ERK and NF-κB pathways, inducing the release of pro-inflammatory chemokines (such as CCL20 and CXCL1) and the pathogenic cytokine IL-23 (4, 12, 22). This mechanism highlights a defining feature of entheseal pathology: stromal cells are not merely structural supports, but immunosurveillance-competent “sentinel cells” that can initiate sterile inflammation solely in response to fluctuations in mechanical load, even in the absence of exogenous pathogens.

Subsequently, stromal-derived IL-23 functions as a central messenger within the local niche, selectively activating specific resident lymphocyte subsets. The entheseal microenvironment-particularly the bone–enthesis interface at the junction of fibrocartilage and vascularized marrow, is enriched with IL-23 receptor-expressing γδ T cells, mucosal-associated invariant T (MAIT) cells, and group 3 innate lymphoid cells (ILC3s). Unlike peripheral T cells that depend on antigen presentation, these resident cell populations remain in a state of continuous readiness for immediate response. In contrast to the degenerative changes observed in IDD, the early activation of these resident innate immune cells and the subsequent IL-23/IL-17 axis cascade in axSpA represent a distinctive hallmark of the immune-driven nature of spinal inflammation (13, 14, 23). This mesenchymal-immune crosstalk not only explains why axSpA inflammation is consistently localized to mechanically stressed sites, but also illuminates the duality of its pathological outcome: IL-17 primarily recruits neutrophils, promoting local inflammatory infiltration and bone erosion, whereas IL-22, by activating STAT3 signalling in MSCs, directly triggers osteogenic differentiation programmes (24). The coexistence of cytokine-partitioned erosion and ossification provides a molecular basis by which mechanical stress is ultimately translated into irreversible structural damage, including syndesmophyte formation.

### 2.2 Intervertebral disc: immune responses under hypoxia and metabolic constraint

Although the intervertebral disc and the enthesis share core mechanosensing mechanisms, such as Piezo1 signaling, their pathological outcomes diverge in fundamentally different directions. This divergence arises because disc degeneration is driven not only by mechanical loading, but also by maladaptive immune-cell responses within a hostile microenvironment. The disc is the largest avascular organ in the human body and relies on diffusion through the endplate to sustain a severely limited nutrient supply, placing it in a state of “physical immune privilege” under steady-state conditions (25, 26). However, when mechanical overload or ageing compromises the integrity of the blood–disc barrier, peripheral monocytes/macrophages rapidly infiltrate the injured region and are abruptly exposed to a metabolic stress environment characterized by hypoxia, high osmolarity, and the accumulation of acidic metabolites. Notably, the physiological hypoxia of the nucleus pulposus (NP) (pO_2_ < 1%) compels resident cells to maintain constitutive stabilization of HIF-1α to sustain aerobic glycolysis—an evolutionary adaptation that supports survival within a low-oxygen niche (16, 27, 28).

For newly infiltrating peripheral macrophages, however, this severe hypoxia represents a major metabolic challenge. In normoxic tissues, macrophages typically retain the plasticity to generate energy via mitochondrial oxidative phosphorylation (OXPHOS) and to repolarize towards a reparative M2 phenotype. Within the disc, by contrast, persistent HIF-1α stabilization forces macrophages into metabolic reprogramming that relies predominantly on the comparatively inefficient Warburg-like programme. Functionally, this shift “locks” macrophages into a pro-inflammatory, M1-like state: intermediates of glycolysis and the truncated tricarboxylic acid cycle-particularly succinate and fumarate—accumulate intracellularly and, by inhibiting prolyl hydroxylase (PHD) activity, sustain transcriptional activation of inflammatory genes such as IL-1β at the epigenetic level (29–31). This metabolic “locking” effect helps explain why inflammation in degenerative discs is often chronic and non-resolving macrophages lose the capacity to transition towards anti-inflammatory, tissue-repair phenotypes.

Sustained aerobic glycolysis is accompanied by marked lactate accumulation within the anatomically confined disc space, driving local pH to below 6.0. This acidic microenvironment is not merely a metabolic by-product but a key pathogenic signalling mediator. On one hand, extracellular acidosis activates acid-sensing ion channels (ASICs, particularly ASIC3) and transient receptor potential (TRP) channels on peripheral terminals of dorsal root ganglion (DRG) neurons, triggering cation influx and directly promoting nociceptive sensitization. Moreover, studies have shown that chemical stimuli originating from the NP can retrogradely induce NF-κB activation in DRG neurons, upregulating ASIC3 expression and lowering mechanical pain thresholds offering a molecular explanation for the frequently observed “symptom-structure dissociation,” in which clinical pain severity does not neatly track radiographic degeneration (32, 33). On the other hand, high lactate concentrations reshape intracellular metabolic flux in infiltrating T cells via monocarboxylate transporters (such as SLC16A1), markedly suppressing the survival and immunosuppressive function of regulatory T (Treg) cells while enhancing the pathogenic capacity of effector T cells to produce IL-17 (28, 34). This metabolically imposed immunoregulatory failure effectively undermines intrinsic resolution programmes within the disc, entrapping the tissue in a vicious pathological cycle marked by excessive expression of catabolic enzymes, including a disintegrin and metalloproteinase with thrombospondin motifs (ADAMTS) and matrix metalloproteinases (MMPs).

### 2.3 Cartilage endplate: a “metabolic–immune interaction hub” linking bone marrow and the intervertebral disc

Beyond the enthesis and the nucleus pulposus, the cartilage endplate constitutes a third defining dimension of the spinal microenvironment. Serving as both a physical barrier and a nutritional gatekeeper between vertebral bone marrow and the avascular intervertebral disc, endplate integrity directly determines the physiological homeostasis of the spinal unit. During disc degeneration, pathological changes of the endplate are dominated by aberrant calcification and loss of permeability. Microfractures induced by mechanical loading frequently trigger post-traumatic repair and sclerotic remodelling, which not only occlude the principal route for glucose and oxygen diffusion into the nucleus pulposus, but also markedly exacerbate metabolic deprivation within the disc, thereby establishing a vicious cycle (35).

More importantly, the endplate represents a key site mediating pathological crosstalk between the bone marrow and the intervertebral disc. Evidence suggests that aberrant endplate calcification is a critical pathological feature driving this interaction. Calcification not only increases endplate stiffness, but also alters the segmental biomechanical environment and induces local tissue inflammation. Radiologically, this process is often reflected by Modic changes, a characteristic feature of IDD (36). When the endplate barrier is compromised or ruptured, its immune-isolating function is lost, allowing pathological contact between immune cell–rich marrow and the otherwise immune-privileged nucleus pulposus. This barrier failure, in turn, promotes ectopic neovascularization, creating a biological conduit for marrow-derived macrophages and T cells to infiltrate the disc interior (37, 38). In addition, pro-inflammatory mediators within the marrow niche can diffuse through the damaged endplate into the nucleus pulposus, activating catabolic programmes in resident cells. Thus, the cartilage endplate is not merely a valve that regulates metabolic flux; it is a pivotal pathological hub that enables inflammatory processes to traverse from bone marrow into the intervertebral disc.

## 3 The core drivers: driving mechanisms of chronic spinal inflammation

The sustained evolution of chronic spinal inflammation is not a simple signalling cascade; rather, it represents a complex biological programme driven by genetic susceptibility, shaped by perturbations in intracellular homeostasis, and ultimately expressed as bone remodelling and neural sensitization. This multidimensional set of drivers ensures the persistence of inflammation within spinal niches and orchestrates pathological restructuring of tissue architecture (39, 40).

### 3.1 Coupling genetic susceptibility to intracellular stress: the pathogenic effects of HLA-B27

HLA-B27 is the central genetic signature of axSpA, and its pathogenic role extends beyond conventional antigen presentation. The main competing hypotheses regarding its disease mechanisms include misfolding-induced unfolded protein response (UPR), formation of cell-surface free heavy-chain homodimers, and aberrant presentation of arthritogenic peptides. Together, these mechanisms contribute to extensive remodeling of intracellular homeostasis and the local immune microenvironment.

At the level of endoplasmic reticulum stress, the high propensity of the HLA-B27 heavy chain to misfold during biosynthesis can directly induce sustained UPR activation. This intracellular stress response enhances IL-23 production by bone marrow-derived cells through activation of the transcription factor XBP1s, thereby providing persistent signaling pressure on the IL-23/IL-17 axis (41–43). Beyond immune activation, XBP1 splicing also contributes to local pathological osteogenesis. In mesenchymal stem cells derived from the enthesis of patients with AS, HLA-B27-associated endoplasmic reticulum stress induces sXBP1 generation, which subsequently activates tissue-nonspecific alkaline phosphatase (TNAP) by upregulating retinoic acid receptor β (RARB). This sXBP1-TNAP signaling axis has been shown to bypass canonical skeletal developmental programs and drive pathological mineralization independently of the core osteogenic transcription factor Runx2, thereby accelerating aberrant matrix mineralization and syndesmophyte formation (44). In addition, HLA-B27 misfolding is often accompanied by impaired autophagic clearance, leading to intracellular accumulation of dysfunctional mitochondria and other stress-related components, which can trigger endogenous danger signals (45). This stress state interacts closely with the mechanical microenvironment of the spine. Highly expressed mechanosensitive YAP1 can inhibit ubiquitin-mediated degradation of the NLRP3 inflammasome, thereby amplifying the release of IL-1β and IL-17A (46). The regulatory network formed by autophagy defects and aberrant mechanotransduction drives a chronic self-sustaining “stress–inflammation” cycle, even in the absence of exogenous pathogens (47).

At the level of cell-surface interactions, aberrant HLA-B27 presentation constitutes a non-canonical pro-inflammatory mechanism. HLA-B27 heavy chains can form β2-microglobulin-free homodimers on the cell surface (48). These abnormal dimers are specifically recognized by the immunoglobulin-like receptor KIR3DL2 on natural killer cells and Th17 cells. Through upregulation of the anti-apoptotic factor Bcl-2 and activation of the transcription factor RORγt, this interaction directly promotes the aberrant differentiation, expansion, and long-term survival of pathogenic Th17 cells (49). This signaling pattern, which extends beyond the conventional function of MHC class I molecules, helps explain the persistent Th17 bias observed in spinal inflammation.

At the level of canonical antigen presentation, recent studies have revealed a direct pathogenic role for arthritogenic peptides. HLA-B27 can present specific pathogenic peptides that selectively activate and drive clonal expansion of autoreactive CD8^+^ T cells expressing the TRBV9 receptor. Clinical evidence indicates that monoclonal antibody-mediated depletion of these pathogenic memory TRBV9^+^ CD8^+^ T cells can induce sustained disease remission and significantly improve spinal mobility parameters and BASMI scores (50). Together with the effects of endoplasmic reticulum aminopeptidase 1 (ERAP1) polymorphisms on peptide trimming (51), HLA-B27 appears to establish a dual pro-inflammatory engine: intracellularly through UPR activation and autophagy disturbance, and at the cell surface through receptor-mediated immune interactions. This dual mechanism places local spinal immune cells in a primed inflammatory state that can be readily amplified by physical stress.

Notably, this local hyper-responsiveness does not occur in isolation, but is influenced by the gut-spine axis. Studies suggest that HLA-B27 and microenvironmental stress-driven gut dysbiosis can induce sustained intestinal IL-23 secretion by Paneth cells and macrophages (42, 52). Elevated IL-23 levels promote inflammatory priming and expansion of lymphocytes expressing the gut-homing integrin α4β7, particularly γδ T cells, MAIT cells, and NKp44^+^ ILC3s. These sensitized cells then enter the circulation and preferentially home to spinal entheseal and adjacent bone marrow sites that highly express the corresponding ligand MAdCAM-1. Clinical evidence showing the simultaneous presence of these homing cell populations in the gut, synovial fluid, and pathological entheses of patients with axSpA provides direct human support for an immune model of gut priming followed by bone-directed migration (14, 53–55).

Clarifying this multidimensional pathogenic network also helps explain a key clinical observation in axSpA: why IL-17 targeted therapies remain effective in HLA-B27-negative patients (56). This finding suggests that IL-17 is not driven solely by a single genetic background, such as HLA-B27, but rather represents a final common pathway converging from multiple upstream pathological signals. Whether initiated by local aberrant mechanotransduction or by systemic immune dysregulation along the gut-joint axis, these non-MHC-restricted pathogenic routes can ultimately converge on explosive local IL-17 release within the spine.

### 3.2 Context-dependent signaling: molecular basis of spinal pathological divergence

One of the most challenging questions in spinal pathology is why comparable mechanical stress and inflammatory exposure drive ankylosing ossification at the enthesis, yet precipitate tissue dissolution and collapse in the adjacent intervertebral disc. Mechanistic dissection indicates that this paradox is not rooted in intrinsic differences in upstream signalling molecules; rather, it reflects lineage-specific epigenetic landscapes that differentially integrate common pathway inputs.

#### 3.2.1 Context-dependent mechanotransduction: the dual faces of YAP/TAZ

As central effectors that sense physical stress, YAP and TAZ are robustly activated both in mechanically loaded entheses and in degenerating discs, yet the biological outcomes are diametrically opposed. Within the entheseal niche, resident MSCs exhibit a chromatin accessibility landscape that is preconfigured towards osteogenesis. Driven by high tensile forces, nuclear YAP preferentially cooperates with transcription factors to activate enhancers controlling osteogenic regulators and genes such as type I collagen, thereby directly translating physical stress into bone-forming signals (57–59).

By contrast, in the nucleus pulposus, resident cells are predominantly notochord-derived NPCs. Lacking epigenetic permission for osteogenic programmes, nuclear accumulation of YAP fails to initiate bone formation and instead engages in pathological molecular interactions. Authoritative work has shown that, in chondrocyte-like lineages, aberrantly activated YAP can form transcriptional complexes with the key NF-κB component p65 (60, 61). This non-canonical binding mode hijacks YAP’s transcriptional activity, markedly amplifying expression of catabolic genes, particularly MMP13 and ADAMTS5, thereby driving irreversible matrix degradation. Consequently, YAP exhibits context-dependent functional duality: executing the osteogenic program at the enthesis while driving matrix decomposition in the IVD.

#### 3.2.2 Spatial heterogeneity of Wnt signalling and failure of the loss of negative feedback control

Differences in the outcomes of canonical Wnt/β-catenin signalling across these two pathological settings further underscore microenvironmental heterogeneity. In degenerative discs, although Wnt ligands are markedly upregulated, the consequences are profoundly destructive. Because nucleus pulposus cells suppress osteogenic potential during development to maintain a highly hydrated matrix, forced activation of Wnt signalling does not drive osteogenic differentiation; instead, it triggers senescence programmes. Excessive Wnt activity has been shown to induce senescence-associated β-galactosidase and matrix-degrading enzymes (62), leading to cell-cycle arrest and the release of abundant senescence-associated secretory phenotype factors, thereby accelerating degeneration rather than regeneration.

At the enthesis in axSpA, by contrast, the pathological core lies in a local loss of Wnt negative-feedback control. Studies have confirmed that, in spinal lesions from patients with this disease, the expression of sclerostin, a Wnt antagonist secreted by osteocytes, is nearly abolished. Moreover, reduced serum levels of this antagonist are significantly negatively correlated with the formation of syndesmophytes (63–65). Disruption of this regulatory mechanism leads to aberrant activation of the Wnt signaling pathway in entheseal mesenchymal cells, thereby forcibly initiating a pathological osteogenic program.

Critically, activation of this osteogenic programme often acquires autonomy, consistent with the clinically observed uncoupling between inflammatory suppression and structural progression. This autonomy arises because the local niche imprints durable pathogenic memory through epigenetic mechanisms. First, high levels of TNF-α can induce METTL14-dependent m6A modification in AS-derived MSCs, stabilizing expression of pathogenic genes such as ELMO1 and conferring aberrant migratory and osteogenic capacity that persists even after removal from an inflammatory milieu (66). Second, once MSCs are recruited, pathological osteogenic differentiation is further governed by the IL-23/IL-17 axis. IL-22 has been shown to act directly on recruited MSCs, inducing osteogenesis-related gene expression and promoting differentiation towards an osteogenic lineage (13, 24). Recent single-cell transcriptomic evidence further reveals that stromal cells within the entheseal niche have undergone lineage phenotypic transition, giving rise to a highly pathogenic Osteo-CAR subpopulation that, through interactions with CD99^+^ neutrophils, collectively locks the osteogenic programme into an autonomous state at both molecular and cellular levels (67). This multilayered locking from N6-methyladenosine modification to the emergence of specific pathogenic cell subsets, helps explain why anti-inflammatory therapy alone rarely reverses structural progression.

Taken together, the divergent structural fates of ankylosing spondylitis and intervertebral disc degeneration reflect the interplay between intrinsic epigenetic memory and extrinsic signalling inputs. Osteogenic permission in entheseal MSCs, versus osteogenic confinement in nucleus pulposus cells, determines whether the same mechano-inflammatory storm culminates in pathological ankylosing bony bridges or in degenerative matrix collapse.

### 3.3 Neuro-immune interplay: a pathogenic loop linking pain and inflammation

Neuro-immune crosstalk represents another core dimension driving spinal microenvironmental destabilization, directly coupling clinical pain with the trajectory of tissue pathology. In degenerative tissues, the nervous system has shifted from a passive nociceptive sensor to an active immunoregulatory unit that participates in reshaping the local niche. Studies have demonstrated pronounced ectopic ingrowth of sensory nerve fibres and accumulation of neuropeptides within degenerating intervertebral discs, providing a biological basis for the persistence of neurogenic inflammation (68).

Mechanistic studies indicate that upregulated secretion of endogenous neurotransmitters such as calcitonin gene–related peptide (CGRP) can profoundly modulate local cellular states through engagement of its receptor component, receptor activity–modifying protein 1 (RAMP1). Although the CGRP-RAMP1 axis is essential for maintaining matrix homeostasis under physiological conditions, in pathological contexts excessive CGRP activates MAPK and NF-κB signalling, markedly inducing apoptosis of NP cells and amplifying the synthesis of pro-inflammatory mediators (69, 70). Moreover, neurogenic signals, through crosstalk with infiltrating macrophages, can reprogram their immune-response phenotypes, shifting spinal inflammation from a self-limited defensive reaction to a self-reinforcing neuro-immune pathological loop (68, 71). This interaction framework not only helps explain the clinical intractability of chronic back pain, but also reveals a deeper biological basis for irreversible disruption of microenvironmental homeostasis.

This neurogenic inflammation further drives pathological neurovascular coupling. The intervertebral disc is physiologically avascular. However, stimulation by pro-inflammatory factors, together with proteoglycan loss, disrupts the integrity of the local microenvironmental barrier. As a result, newly formed blood vessels and sensory nerve fibers concomitantly infiltrate into the nucleus pulposus (72, 73).

These neostructures fundamentally remodel local ecology: newly formed vessels provide metabolic support for sustained recruitment of inflammatory cells, whereas newly ingrown sensory endings, via high expression of ASICs such as ASIC1a and ASIC3, become exquisitely sensitive to lactate accumulation and low pH in the degenerative milieu (32, 74). Evidence suggests that ASIC activation not only directly mediates discogenic low back pain, but also promotes pro-inflammatory effector programmes and accelerates apoptosis, thereby forming a positive-feedback cascade with inflammatory mediators produced by immune cells. This interaction pattern is characterized by inflammation-induced nerve ingrowth. In turn, neural signaling feeds back to amplify inflammation. Together, these processes transform spinal inflammation from a localized response into a self-reinforcing pathological program (73, 74). The supporting evidence for the core mechanisms described above is summarized in **Table 1**.

**Table 1:** Summary of evidence grading for core mechanisms.

Core mechanism	Summary of mechanisms	*In vitro* evidence	*In vivo* evidence	Human/clinical data	Reference
Mechano-inflammation	Piezo1-mediated Ca^2+^ influx senses physical stress, triggering pathological entheseal osteogenesis and intervertebral disc matrix degradation.	Murine BMDMs/monocytes under cyclical hydrostatic pressure (CHP); Osteocytes under fluid shear stress.	Myeloid-specific *Piezo1* knockout mice (autoinflammation model); Osteoblast-specific *Piezo1* conditional knockout mice.	—	(19, 20)
Metabolic reprogramming	Hypoxia-induced Warburg effect leads to succinate accumulation, stabilizing HIF-1*α* via PHD inhibition and locking the pro-inflammatory M1 phenotype.	Metabolic flux analysis and transcriptional profiling of LPS-stimulated murine BMDMs and human PBMCs.	LPS-induced murine sepsis model; *B. pertussis* or *S. typhimurium* infection models.	—	(29–31)
Epigenetic Memory	H3K27ac modifications form super-enhancers, recruiting BRD4 to maintain autonomous transcriptional imprints of osteogenic genes (e.g., *ZBTB16*).	H3K27ac/BRD4 ChIP-seq and dual-luciferase reporter assays in human BM-MSCs.	Mesenchymal-specific *Brd4* conditional knockout mice (*Prx1-cre*); Local application of JQ1 to block bone repair.	Validation in primary MSCs from osteoporosis patients, confirming altered expression profiles of BRD4 and super-enhancer target genes.	(113–115)
sXBP1-TNAP Axis	HLA-B27 misfolding induces UPR, activating the sXBP1-RARB axis to upregulate TNAP and drive Runx2-independent mineralization.	Osteogenic induction, shRNA interference, and HLA-B27 lentiviral overexpression in human AS-MSCs.	Orthotopic implantation of AS-MSCs in NOD-SCID mice (lumbar injury model); Systemic TNAP inhibitor intervention.	Serum BAP validation in Taiwanese and British cohorts; TNAP overexpression in AS patient bone marrow and entheseal MSCs.	(44)
Gut-Joint Axis	Gut dysbiosis drives enteric IL-23 secretion, promoting *α*4*β*7^+^ ILC3 expansion and homing to mechanical-loading entheses.	Co-culture induction of human LTi cells with AS patient intestinal epithelial cells.	Germ-free HLA-B27 transgenic rats; SKG mouse model of IL-23-dependent gut-joint pathology.	IL-23 expression in AS patient ileal biopsies; Flow cytometry confirming *α*4*β*7^+^ ILC3 expansion in gut/blood/bone marrow and ILC3 presence at the enthesis.	(14, 42, 53)

## 4 Therapeutic strategies: precision targeting and niche reprogramming in the spine

As understanding deepens that spinal pathology is driven by the local microenvironment, therapeutic perspectives are expanding beyond the simple suppression of downstream inflammatory mediators toward coordinated remodeling of upstream niches. Such integrated strategies may enable deeper disease modification by targeting the mechano-inflammatory cycle, releasing metabolic reprogramming, and correcting aberrant transcriptional programs (75, 76).

On this basis, restoring immune homeostasis further requires a spatiotemporally stratified treatment framework. This entails precisely identifying the inflection point at which AS transitions from an immune storm phase to an autonomous osteogenesis phase, and the trajectory by which IDD evolves from a “metabolic adaptation” phase into a “senescence and failure” phase. Accordingly, the strategies proposed in this section are not isolated target interventions, but dynamic regimens aligned to these pathological windows: early-stage precision immune/metabolic blockade, followed in later stages by epigenetic modulation and mechanical signal unloading (**Table 2**).

**Table 2:** A intervention map for chronic inflammatory spinal diseases: from metabolic reprogramming to epigenetic rewriting.

Clinical intervention window	Strategy	Representative agents	Molecular targets	Signaling pathways & cascades	Spine-specific pathological impacts	Reference
Early-stage axSpA: Immune-driven Phase	Immune Interception & Niche Protection	Biologics (e.g., Secukinumab); Triptolide	TNF-*α*, IL-17A, JAK2, STAT3	Blockade of the IL-23/IL-17 axis; inhibition of JAK2 phosphorylation and subsequent STAT3 activation.	Quenching the pathogenic activity of resident *γδ* T cells at the enthesis; preventing early inflammatory “education” of MSCs.	(91, 94, 95)
Late-stage axSpA: Structural Remodeling Phase	Epigenetic Memory Erasing	JQ1 (BET inhibitor); SIRT1 activators; miR-29 mimics	BRD4, H3K27ac (Super-enhancers), HDAC4, METTL14	Disruption of BRD4 binding to H3K27ac-rich super-enhancers; resetting the transcriptional landscape via the SIRT1/FOXO1 axis; inhibiting m6A-mediated ELMO1 stability.	Erasing the persistent “osteogenic imprint” on MSC chromatin; resolving the “inflammation-osteogenesis uncoupling” paradox; blocking autonomous new bone formation in the absence of systemic inflammation.	(113, 114) (115)
Early IDD: Metabolic Adaptation Phase	Immunometabolic Shunting	4-Octyl Itaconate (4-OI); TEPP-46 (PKM2 activator)	SDH (Succinate Dehydrogenase), KEAP1/Nrf2, PKM2 (Tetramerization)	Inhibition of SDH to reduce succinate accumulation; activation of Nrf2 antioxidant pathways; preventing dimeric PKM2 nuclear translocation as a co-activator.	Blocking HIF-1*α* pseudohypoxia-induced activation triggered by succinate; releasing macrophages from “pro-inflammatory metabolic locking” in hypoxic niches; inducing M2-like repolarization.	(98, 99)
Late IDD: Senescence & Exhaustion Phase	Niche Purification & Mitochondrial Quality Control	Senolytics (D+Q: Dasatinib + Quercetin); Melatonin	BCL-xL, NLRP3 inflammasome, PPAR*γ*	Interfering with anti-apoptotic pathways in senescent cells; inducing mitophagy; inhibiting oxidized mtDNA release and subsequent cGAS-STING activation.	Clearing senescent nucleus pulposus cells (SASP sources); reducing endogenous damage-associated molecular patterns (DAMPs) released by damaged mitochondria; breaking the mtDNA-triggered inflammatory loop.	(100, 103, 104)
Global: Physico-chemical Transduction Phase	Mechano-therapeutics (Molecular Unloading)	YAP inhibitors (e.g., Verteporfin); AMPK activators (e.g., Metformin)	YAP1/TAZ, Piezo1, ASIC1/3, TRPV1	Blocking the pathogenic interaction of YAP with TEAD (osteogenesis) or NF-*κ*B (catabolism); inhibiting YAP nuclear translocation via AMPK; suppressing ion flux from acid-sensing channels.	Achieving “molecular unloading”: blocking the “stress-inflammation-ossification” cascade in the enthesis and the “acidosis-pain-catabolism” feedback loop in the disc without restricting physical loading	(4, 12, 22, 46, 108)
Local Delivery Systems	Microenvironment-responsive Release	MMP-sensitive hydrogels; Functionalized nanoparticles (NPs)	MMP-9, local acidic pH, enzymatic degradation	Utilizing high expression of MMPs or low pH in the enthesis/disc as chemical triggers for controlled and demand-based drug release.	Overcoming the “blood-spine barrier” and diffusion limits in avascular zones; maintaining therapeutic concentrations in deep enthesial tissues; minimizing systemic toxicity from chronic administration.	(92, 93)

### 4.1 Blocking the entheseal IL-23/IL-17 axis: from systemic suppression to locally precise delivery

Currently, biologic DMARDs (bDMARDs) and targeted synthetic DMARDs (tsDMARDs) have become the cornerstone of axSpA treatment (77). This therapeutic landscape has been established largely through a series of landmark clinical trials, including RAPID-axSpA for TNF inhibitors (78), MEASURE for IL-17A inhibition (7), BE MOBILE 1/2 for dual IL-17A/F inhibition (79), and SELECT-AXIS for JAK inhibition (80).

These studies have shown that sustained control of inflammation can significantly improve symptoms and slow radiographic progression of the spine. For example, a Cochrane review of 21 randomized controlled trials reported high-certainty evidence that TNF inhibitors, including adalimumab, etanercept, golimumab, and infliximab, are effective in inducing ASAS40 responses, reflecting composite improvement in pain, function, and inflammation. In addition, both secukinumab and adalimumab have been shown to maintain low levels of spinal radiographic progression over 104 weeks of treatment (81, 82).

However, important limitations remain in clinical practice. Some patients show primary non-response, whereas others develop secondary loss of efficacy due to anti-drug antibodies, such as anti-infliximab antibodies, which substantially limits drug persistence (83). Moreover, clinical data suggest that TNF inhibitors have a marked “lag effect” in structural protection, usually requiring at least 4 years of continuous treatment before inhibition of new bone formation can be radiographically confirmed. In addition, there is currently no conclusive evidence that IL-17A inhibitors, represented by secukinumab, can significantly block radiographic progression within shorter periods such as 2 years (84).

This dissociation between anti-inflammatory effects and structural improvement may be partly explained by the difficulty of maintaining effective biological concentrations of biologics within local spinal niches, particularly at the avascular and mechanically complex entheseal sites (85). Moreover, the failure of the IL-12/23 inhibitor ustekinumab to meet expected endpoints in axSpA clinical trials further reflects the complexity of the entheseal microenvironment. Studies have shown that specific resident immune-cell populations at this site can produce IL-17 independently of IL-23 signaling. This autonomy of local pathological pathways may limit the therapeutic benefit of targeting a single circulating cytokine axis alone (86–88). In addition, the risks of infection and cardiometabolic complications associated with long-term systemic administration may further restrict the clinical potential of these agents (89, 90).

In response to these challenges, therapeutic strategies are increasingly shifting toward intervention during the early immune-driven phase of disease. Evidence suggests that activation of the IL-23/IL-17 axis occurs mainly at disease initiation. This axis not only induces systemic inflammation but also triggers tissue-remodeling programs during the preclinical stage (91). Therefore, within the therapeutic window before autonomous osteogenesis has been established, blockade of this axis may suppress the pathogenic activity of resident immune cells, such as γδ T cells, and thereby prevent aberrant osteogenic differentiation of mesenchymal stem cells induced by prolonged exposure to an inflammatory microenvironment.

To overcome the physical constraints imposed by the blood-spine barrier, the development of local delivery systems based on smart biomaterials has emerged as a frontier direction. Clinical biomarker studies have shown that disease activity in AS correlates significantly with levels of matrix metalloproteinases such as MMP-9 (92). MMP-responsive hydrogels or functionalized nanoparticles can therefore be engineered to achieve microenvironment-triggered release specifically within entheseal lesions (93). These local platforms can further be loaded with multi-target small molecules such as triptolide. Triptolide has been shown to markedly attenuate inflammatory progression in axSpA by inhibiting key pathways including JAK2/STAT3 (94), moreover, such agents can modulate the biological properties of MSCs, thereby restraining pathological dysregulation of bone metabolism at its source (95). Compared with large monoclonal antibodies, these small molecules exhibit superior tissue penetrance, enabling more effective diffusion into the deep entheseal microenvironment where blood supply is sparse and mechanical stresses are complex. T This strategy can not only efficiently suppress local immune responses but also synchronously block pro-inflammatory cytokine production and pathogenic new bone formation by selectively inhibiting STAT3 phosphorylation. In this way, it maximizes local therapeutic efficacy while minimizing systemic toxicity (96).

### 4.2 Metabolic reprogramming within the intervertebral disc: optimizing glycolysis and mitochondrial quality control

Given the hypoxic nature of the intervertebral disc microenvironment, blindly reactivating highly oxygen-consuming OXPHOS may not be an optimal strategy. Potential dysfunction of the electron transport chain may even trigger a burst of ROS production. This suggests that immunometabolic remodeling may be achieved through adaptive adjustment of metabolic pathways and fine regulation of mitochondrial homeostasis. The central premise is to promote macrophage transition towards reparative phenotypes through shifts in substrate preference (for example, enhancing fatty-acid oxidation) without increasing oxygen demand, while simultaneously limiting endogenous danger signalling by clearing damaged mitochondria (97).

First, targeted modulation of the “glycolysis-inflammation” axis is pivotal for restoring immune stability. Pro-inflammatory macrophages rely heavily on the Warburg programme, leading to pathological breaks in the tricarboxylic acid cycle and accumulation of the pro-inflammatory metabolite succinate. Itaconate derivatives (such as 4-octyl itaconate, 4-OI) play a central regulatory role in this context. Evidence shows that 4-OI activates the Nrf2 antioxidant pathway by alkylating KEAP1 and selectively inhibits succinate dehydrogenase (SDH). Rather than forcibly restoring mitochondrial ATP production, this mechanism interrupts the conversion of succinate to fumarate, thereby preventing succinate-driven stabilization of HIF-1α and subsequent IL-1β release (98). In parallel, interventions targeting PKM2 require precise tuning: allosteric activators (such as TEPP-46) promote tetramerization of PKM2, principally to prevent its nuclear translocation in the dimeric form and thereby block its non-metabolic role as a transcriptional cofactor for STAT3 or HIF-1α. The goal is to suppress inflammatory cascades at the transcriptional level, rather than simply to restore mitochondrial energetics under hypoxia (99).

Second, preserving mitochondrial integrity to limit mtDNA leakage offers another feasible anti-inflammatory route in hypoxic settings. In degenerative discs, oxidized mtDNA released from damaged mitochondria is a potent endogenous agonist of the NLRP3 inflammasome (100). Recent studies indicate that activating PPARγ-dependent pathways can markedly attenuate oxidative stress–induced mitochondrial injury (101), or that melatonin can induce mitophagy (100), thereby selectively eliminating dysfunctional mitochondria and suppressing inflammation. This approach not only prevents excessive ROS accumulation, but also restores intracellular metabolic homeostasis, effectively supporting the survival and function of reparative cells under low oxygen tension.

Meanwhile, senolytic strategies targeting senescent cells that accumulate during IDD show considerable therapeutic potential. Under sustained oxidative stress and chronic inflammatory stimulation, degenerated nucleus pulposus cells (NPCs) often enter a senescent state through DNA damage responses and acquire a senescence-associated secretory phenotype (SASP). These cells continuously release matrix metalloproteinases and pro-inflammatory mediators, thereby acting as an endogenous pathological source of chronic inflammation within the degenerative disc microenvironment (102, 103). Experimental evidence in recent years shows that the D+Q combination or agents targeting the BCL-xL pathway can selectively disrupt senescent cells’ anti-apoptotic circuits and induce their preferential apoptosis (103, 104). This microenvironmental purification strategy not only blocks the source of pathological factors but also markedly improves local biological properties. In doing so, it creates an optimized microenvironmental niche for endogenous stem cell homing and tissue repair (11, 102).

### 4.3 Mechanotransduction-targeted therapies: intercepting mechano-inflammatory transduction at its source

Physical loading is a primary initiator of spinal inflammation, and microdamage within stress-concentrated entheseal regions, coupled to inflammation, is central to disease onset (105). Given the clinical reality that spinal load-bearing cannot be fully avoided, the concept of mechanotherapeutics has gained traction. This approach does not refer to conventional physical therapy; rather, it aims to block cellular responses to mechanical stress by pharmacologically intervening in intracellular mechanotransduction pathways (106).

YAP1, a core effector that senses mechanical tension and changes in matrix stiffness, is a key therapeutic target. Studies have shown pathological overexpression of YAP1 in spinal tissues from patients with AS, a process driven by mechanical stress and finely regulated by epigenetic mechanisms. For example, the long non-coding RNA HULC upregulates YAP1 by competitively binding miR-556-5p, thereby inducing chondrocyte apoptosis and inflammatory responses (107). Activated YAP1 further suppresses ubiquitin-dependent degradation of the NLRP3 inflammasome, stabilizing the complex and promoting release of pathogenic mediators such as IL-17A and IL-23 (46). In addition, YAP1 activation is closely linked to pathological osteogenesis in the spine. Through crosstalk with Wnt/β-catenin signalling, YAP1 can downregulate Wnt inhibitory factors such as DKK1 and SOST, releasing constraints on osteogenic programmes and driving new bone formation (46, 108). As AMPK is a central sensor of cellular energy metabolism and negatively regulates YAP1 activity, activation of the AMPK pathway represents a potential strategy to interrupt this pathological process. Studies have shown that activated AMPK can directly phosphorylate YAP1 at Ser61 or Ser94, thereby disrupting its interaction with the transcription factor TEAD. In parallel, AMPK can phosphorylate and stabilize the adaptor protein AMOTL1 at Ser793, promoting cytoplasmic retention and inactivation of YAP1 (109, 110). This multilayered inhibitory effect supports the potential use of AMPK activators, such as metformin, to modulate shared mechano-inflammatory pathways at the molecular level.

### 4.4 Epigenetic intervention: selectively intercepting pathological ossification programmes

To address the late-stage uncoupling between anti-inflammatory efficacy and ongoing ossification in AS, therapeutic strategies must extend into the level of transcriptional governance. Targeting the epigenetic and metabolic remodelling programmes that drive ossification is pivotal for reversing this otherwise intractable phase. Chronic inflammation imprints durable epigenetic memory in MSCs, and the highly conserved deacetylase SIRT1 acts as a multidimensional regulatory hub in this process.

Evidence indicates that the FOXO1-SIRT1 axis integrates immunometabolic cues and oxidative stress signals within the local niche, thereby directing spinal bone homeostasis through epigenetic regulation (111). Dysregulation of this axis in the pathological milieu of AS not only exacerbates metabolic disturbance but also releases transcriptional repression over a suite of pro-osteogenic genes. In parallel, genetic susceptibility can lock immune-cell pathogenicity through epigenetic mechanisms. For example, epigenetic modifications driven by TBX21 polymorphisms sustain IL-17 and IFN-γ expression in pathogenic CD8^+^ T cells and natural killer cells; this persistent cytokine pressure leaves permissive osteogenic marks on stromal-cell chromatin (112).

More importantly, histone modifications, particularly H3K27ac, are key determinants of MSC differentiation fate. Studies have shown that, in MSCs derived from patients with osteoporosis, H3K27ac-marked super-enhancers recruit BRD4 to maintain an aberrant osteogenic gene-expression program. This mechanism has been validated *in vivo* using Prx1-Cre–mediated Brd4 knockout mice (113). In the entheseal microenvironment of AS, prolonged inflammatory stimulation can hijack this physiological programme, inducing durable super-enhancers at osteogenesis-related genes. Even after withdrawal of inflammatory mediators, these H3K27ac-enriched chromatin regions can remain accessible through BRD4, thereby encoding an “osteogenic memory”. Accordingly, disrupting BRD4 super enhancer interactions with BET inhibitors such as JQ1 has emerged as a leading strategy to block pathological ossification and reverse the uncoupling phenomenon (114). In addition, combining miRNA-based therapies may further reset these pathogenic imprints. Evidence indicates that m6A methylation regulates the miR-29b-3p/HDAC4 axis to precisely determine MSC lineage commitment; restoring suppressed miR-29 expression can specifically target collagen biosynthetic pathways and inhibit HDAC4, thereby reversing aberrant ossification driven by pathogenic memory at the gene-regulatory level (115).

### 4.5 Barriers to clinical translation: from preclinical evidence to standardized evaluation systems

Although targeting upstream microenvironmental regulators, including metabolic checkpoints, Piezo1/YAP-TAZ mechanotransduction, and epigenetic modulators, has shown potential to modify pathological processes in preclinical models, translation into human disease remains limited by insufficient tissue specificity and potential off-target effects. Systemic inhibition of mechanotransduction pathways or broad modulation of epigenetic marks may interfere with physiological bone remodeling and systemic immune defense. Therefore, although compounds such as TEPP-46, JQ1, and metformin have demonstrated microenvironment-modulating effects at the preclinical stage, technical barriers related to local and controlled delivery must be overcome before large-scale clinical validation.

Within this translational trajectory, several compounds with potential microenvironment-modulating properties have already entered clinical evaluation or real-world investigation. Small-molecule agents represented by targeted synthetic DMARDs, such as the selective JAK1 inhibitors ivarmacitinib and upadacitinib, may restore local niches by intercepting upstream signaling reprogramming, and have shown significant efficacy with manageable safety profiles in phase 2/3 clinical trials for AS (116, 117). These clinical data provide preliminary support for the feasibility of upstream intervention strategies.

To accurately assess the clinical benefit of “microenvironment remodeling, ” an evaluation system integrating biomarkers with multidimensional clinical endpoints is required. Future study designs should use ASDAS/BASDAI scores, ASAS20/40 response rates, and patient-reported outcomes (PROs) to evaluate improvements in symptoms and quality of life, while linking these measures to imaging endpoints that reflect objective microenvironmental changes. For example, MRI-based SPARCC scores can be used to quantify the resolution of bone marrow edema and monitor reversal of the local inflammatory niche, whereas long-term mSASSS follow-up is needed to confirm the ultimate inhibition of radiographic progression. Integrating these established clinical readouts with niche-specific biomarkers provides a rational framework for determining whether upstream microenvironmental interventions can truly achieve disease modification.

## 5 Conclusions and outlook

The clinical discordance between inflammatory control and structural progression in inflammatory spinal diseases reveals deeper pathological mechanisms that remain insufficiently addressed by conventional therapies. Evidence suggests that the unique anatomical niche of the spine, through multidimensional interactions among mechanical loading, hypoxic metabolism, and genetic susceptibility, induces pathological reprogramming and metabolic reprogramming of immune cells, thereby establishing an intrinsic barrier of resistance to anti-inflammatory agents. Accordingly, clinical intervention strategies are gradually shifting from merely targeting downstream inflammatory mediators toward the coordinated remodeling of the upstream microenvironmental ecosystem. This shift requires the development of a multidimensional precision intervention framework. Pharmacological strategies may be used to block the transduction of mechanical stress into biochemical signals; metabolic modulators may reverse glycolytic skewing and promote inflammatory resolution; and epigenetic interventions may erase pathogenic memory encoded in chromatin. In addition, deep characterization of pathological landscapes by spatial omics, together with the effective penetration of the blood-spinal barrier using intelligent biomaterials, will be critical for realizing this therapeutic vision.

In summary, viewing the spine as an immune–mechanical–metabolic coupling organ provides a new theoretical framework for overcoming refractory structural failure. By precisely reconstructing microenvironmental homeostasis and identifying critical therapeutic windows, it may be possible to interrupt the vicious cycle between chronic inflammation and structural deterioration, ultimately achieving the dual goals of preserving spinal architecture and maintaining patient function.
